# Dispersive Solid-Phase Extraction and Ultra-Performance Liquid Chromatography–Tandem Mass Spectrometry—A Rapid and Accurate Method for Detecting 10 Macrolide Residues in Aquatic Products

**DOI:** 10.3390/foods13060866

**Published:** 2024-03-13

**Authors:** Jinyu Chen, Guangming Mei, Xiaojun Zhang, Daoxiang Huang, Pengfei He, Dan Xu

**Affiliations:** 1 College of Food and Pharmaceutical Science, Zhejiang Ocean University, Zhoushan 316022, China; chenjinyu3355@163.com (J.C.); huangdaoxiang2024@163.com (D.H.); 2 Zhejiang Marine Fisheries Research Institute, Zhoushan 316021, China; xiaojun3627@163.com (X.Z.); fuzhou.pengfei@zjou.edu.cn (P.H.); xdplmm@126.com (D.X.)

**Keywords:** antibiotic residue, macrolide, solid-phase extraction, isotopically labelled internal standard, LC-MS/MS

## Abstract

The amount of macrolide (MAL) residues in aquatic products, including oleandomycin (OLD), erythromycin (ERM), clarithromycin (CLA), azithromycin (AZI), kitasamycin (KIT), josamycin (JOS), spiramycin (SPI), tilmicosin (TIL), tylosin (TYL), and roxithromycin (ROX), was determined using solid-phase extraction and ultra-performance liquid chromatography–tandem mass spectrometry (UPLC-MS/MS). The residues were extracted with 1% ammonia acetonitrile solution and purified by neutral alumina adsorption. Chromatographic separation was completed on an ACQUITY UPLC BEH C_18_ column with acetonitrile–0.1% formic acid aqueous solution as the mobile phase, and mass spectrometry detection was performed by multiple reaction monitoring scanning with the positive mode in an electrospray ion source (ESI^+^). Five isotopically labeled compounds were used as internal standards for quality control purposes. The findings indicated that across the mass concentration span of 1.0–100 μg/L, there was a strong linear correlation (*R*^2^ > 0.99) between the concentration and instrumental response for the 10 MALs. The limit of detection of UPLC-MS/MS was 0.25–0.50 μg/kg, and the limit of quantitation was 0.5–1.0 μg/kg. The added recovery of blank matrix samples at standard gradient levels (1.0, 5.0, and 50.0 μg/kg) was 83.1–116.6%, and the intra-day precision and inter-day precisions were 3.7 and 13.8%, respectively. The method is simple and fast, with high accuracy and good repeatability, in line with the requirements for accurate qualitative and quantitative analysis of the residues for 10 MALs in aquatic products.

## 1. Introduction

**Figure 1:**
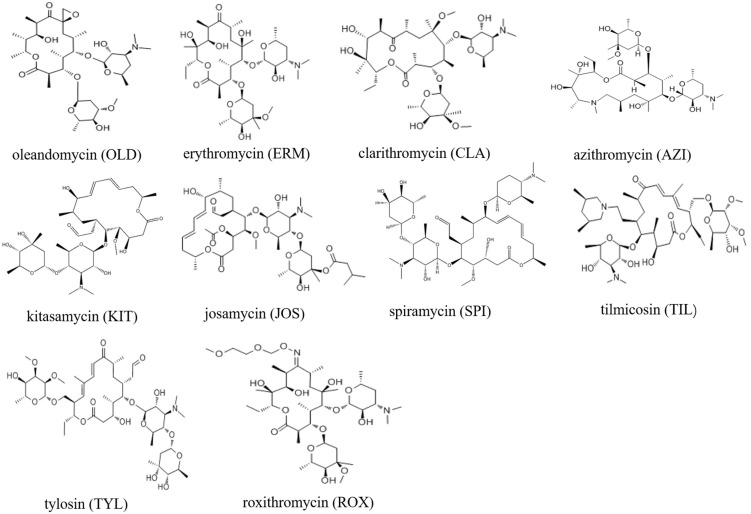
Structures of 10 typical MALs.

Macrolides (MALs) are a group of antibiotics secreted by *Streptomyces*. Structurally, MALs contain a lactonic ring of 12–22 carbon atoms that comprise the core structure, with 1–3 branches of neutral or alkaline sugar chains through glycosidic bonds [1,2]. The structures of 10 typical MALs are shown in Figure 1. MALs show good inhibitory or killing effects on gram-positive bacteria, mycoplasma, and some gram-negative bacteria. Therefore, they are widely applied in treating several diseases in livestock and aquaculture [3,4,5]. In addition, they also improve feed utilization and promote animal growth [6,7].

Excessive use of MALs causes their residues to accumulate in food and animal tissues. Prolonged consumption of livestock and poultry or aquatic food with MAL residues can lead to bacterial resistance and damage human nerves, liver, kidney, and other tissues. Thus, China has clear guidelines on the maximum erythromycin, kitasamycin, spiramycin, tilmicosin, tylosin, and other MAL residue limits in muscles and edible visceral tissues of livestock and poultry. For instance, the residue limit of erythromycin in the muscles and skin of fish is 200 μg/kg [8]. The European Union [9], the United States [10], and Japan [11] have also issued standards for the limit of MAL residues in animal-derived foods. Due to the high aquaculture density and the high risk of eutrophication of water bodies, aquatic animals are susceptible to diseases during the breeding process. Some farmers use antibiotics to treat livestock, poultry, and aquaculture, which increases the risk of MAL contamination of aquatic products. Therefore, there is a need to develop an efficient method for detecting multiple MAL residues in aquatic products, which is key in monitoring the quality and safety of these products.

There are diverse aquatic products with complex matrix composition in aquatic ecosystems. Due to the low content of antibiotic residues in aquatic products, it is necessary to conduct sample pre-treatment to enrich and purify the target before quantitative analysis to improve the sensitivity and reduce interference by non-target compounds. The time consumed by sample pre-treatment usually accounts for more than two-thirds of the whole analytical cycle, which is the key to obtaining accurate results. At present, liquid–liquid extraction (LLE) [12,13,14] and solid-phase extraction (SPE) [15,16,17] are the main pre-treatment and purification methods for determining antibiotic residues in biological samples. SPE removes impurities quickly and effectively and uses fewer organic solvents compared to LLE [18]. Therefore, the SPE method has become the most commonly used pre-treatment technology in drug residue analysis. Based on the principle of conventional SPE, solid-phase microextraction [19,20], magnetic SPE [21], molecularly imprinted SPE [22], and dispersive solid-phase extraction (DSPE) [23,24] have been developed. DSPE has many advantages, such as high efficiency, environmental friendliness, and low cost. It is also very rapid, significantly reducing the analysis time. Thus, given that it meets the needs of food safety analysis, it has been widely used in analyzing multi-drug residues in biological samples [25]. 

At present, thin-layer chromatography [26,27], electrochemical methods [28], fluorescence microscopy [29], microbial titer analysis [30], liquid chromatography [31,32,33,34], gas chromatography-mass spectrometry (GC-MS) [35,36] and liquid chromatography–tandem mass spectrometry (LC-MS) [37,38,39,40] are the main quantitative methods of analyzing MALs in biological samples. LC-MS, which combines the ultra-performance of liquid chromatography with the high sensitivity of mass spectrometry, has become the preferred method for determining the concentration of drug residues in meat products [41]. Currently, the literature on the application of LC-MS in detecting MAL residues in food focuses mainly on livestock and poultry products [2,42], but little attention has been given to aquatic products. In addition, most of the existing MAL detection methods require complicated pre-treatment, only detect a limited number/types of MALs, and have low sensitivity. LC-MS is easily affected by a matrix, and the external standard quantitative method usually has the disadvantages of having a strong matrix effect (ME) and low accuracy. Considering the necessity of detecting MAL residues in aquatic products, this study combined the sample pre-treatment process of matrix DSPE with the LC-MS method to determine multiple MAL residues in aquatic products simultaneously. Furthermore, isotopically labeled compounds were used as internal controls to increase the efficiency, accuracy, and applicability of the method in analyzing the MAL residues in aquatic products.

## 2. Materials and Methods

### 2.1. Materials and Reagents

Erythromycin (ERM) (97.5% purity), clarithromycin (CLA) (98.9% purity), azithromycin dihydrate (AZI) (99.9% purity), spiramycin (SPI) (87.1% purity), tilmicosin (TIL) (84.7% purity), roxithromycin (ROX) (96.5% purity), josamycin (JOS) (91.2% purity), tylosin tartrate (TYL) (82.8% purity), 100 mg/L oleandomycin (OLD) in acetonitrile, and 100 µg/mL kitasamycin (KIT) in acetonitrile were all purchased from the Dr. Ehrenstorfer GmbH company (Augsburg, Germany). Erythromycin-^13^C, D_3_ (ERM-^13^C, D_3_, 95% purity), clarithromycin-N-methyl-^13^C, D_3_ (CLA-N-methyl-^13^C, D_3_, 96% purity), azithromycin-D_3_ (AZI-D_3_, 95% purity), timicosin-D_3_ (TIL-D_3_, 98% purity), and tylosin-D_9_ (TYL-D_9_, 95% purity) were used as internal standards and were purchased from TRC Inc. (Toronto, ON, Canada). Acetonitrile, methanol, and formic acid were of HPLC grade and obtained from the Sigma-Aldrich Corporation (St. Louis, MO, USA). Anhydrous magnesium sulfate and ammonia were analytically pure and purchased from Sinopharm Chemical Reagent Co., Ltd. (Shanghai, China). Neutral alumina (N-Al_2_O_3_, 100–200 mesh, activated), C_18_ (Endcapped, 40–60 μm), and PSA (40–60 μm. 60Å) were purchased from ANPEL Laboratory Technologies Inc. (Shanghai, China). Milli-Q system-purified water was used where necessary.

### 2.2. Instruments and Equipment

An Acquity I-Class UPLC and a Xevo TQ-S triple quadrupole mass spectrometer were purchased from the Waters Corporation (Milford, MA, USA). An MS3D vortex mixer was purchased from IKA (Staufen, Germany). A 5810 desktop centrifuge was purchased from Eppendorf Corporate (Hamburg, Germany). An N-EVAP- 112 nitrogen-blowing instrument was purchased from Organomation (Kansas City, KS, USA). An FJ200-SH high-speed homogenizer was purchased from Hangzhou Jingfei Instrument Technology Co., Ltd. (Hangzhou, China). A FS-2000T ultrasonic processor was purchased from Shanghai Shengxi Ultrasonic Instrument Co., Ltd. (Shanghai, China).

### 2.3. Methods

#### 2.3.1. Standard Solution Preparation

According to their purity and deduction of non-determination parts contained in the molecule (i.e., two water molecules binding to AZI standard and tartrate in TYL), appropriate amounts of eight solid standards (erythromycin, clarithromycin, azithromycin, spiramycin, tilmicosin, roxithromycin, josamycin, and tylosin) were weighed and dissolved in methanol to prepare a single standard stock solution with a concentration of 100 mg/L, while the purchased 100 mg/L standard solutions of OLD and KIT were directly used for subsequent configuration. Appropriate volumes were then accurately taken from each standard stock solution and diluted with methanol to obtain a mixture of 10 standard solutions, with each analyte at a concentration of 0.5 mg/L. The preparations were stored at −18 °C till further use.

Appropriate amounts of five isotopically labeled internal standards, including ERM-^13^C, D_3_, CLA-N-methyl-^13^C, D_3_, AZI-D_3_, TIL-D_3,_ and TYL-D_9_ were weighed according to their purity, dissolved in methanol to prepare an internal standard stock solution with a concentration of 100 mg/L. Appropriate volumes of each internal standard stock solution were accurately taken and diluted with methanol to prepare a mixture of five internal standard solutions, with each analyte at a concentration of 0.5 mg/L. The solutions were stored at −18 °C till further use.

#### 2.3.2. Sample Preparation and Pre-Treatment

The aquatic products for the test were purchased from the Fengmao farmers’ market in Lincheng, Zhoushan City, Zhejiang Province, China between October 2022 and December 2022. The samples used in the experiments of condition optimization and method validation were analyzed in advance to ensure no residual MALs were present. The fish was prepared by removing scales, bones, and visceral organs before taking muscles and skin along the back. The head, shell, and intestinal gland of shrimps were removed, and the muscle was taken. The edible part of the crab was taken. The samples were cut into small pieces of less than 0.5 cm × 0.5 cm × 0.5 cm, homogenized using a high-speed homogenizer, and stored in the refrigerator at −18 °C for subsequent use.

Homogeneous and thawed samples were accurately weighed (5 ± 0.02 g) into a 50-mL polypropylene centrifuge tube before adding 50 μL of 0.5 mg/L mixture of internal standard solution and 20 mL of 1% ammonium hydroxide–acetonitrile solution. After vortexing for 3 min, 20 kHz ultrasonic-assisted extraction was performed for 10 min, followed by centrifugation at 5000 r/min for 5 min. The supernatant was collected into another 50-mL polypropylene centrifuge tube, and 20 mL of 1% ammonium hydroxide–acetonitrile solution was added to the residue for another extraction. After centrifugation, the two supernatants were combined and diluted to 50 mL with acetonitrile. The extract (10 mL) was transferred into a 15 mL polypropylene centrifuge tube, and 1.0 g of anhydrous magnesium sulfate and 2.0 g of neutral aluminum oxide powder were added. After vortexing for 2 min and centrifugation at 5000 r/min for 5 min, all the supernatant was transferred into another 15-mL polypropylene centrifuge tube, placed in a 45 °C water bath, and dried with a gentle nitrogen stream. The test sample was fully dissolved with 1 mL of acetonitrile—0.1% formic acid aqueous solution (*v*/*v*, 5:95) and filtered by a 0.22 μm PTFE microporous filter membrane before analysis with UPLC-MS/MS.

#### 2.3.3. Analysis Parameters of Instruments

Chromatographic condition: Acquity UPLC BEH C_18_ column (2.1 mm × 100 mm, filler particle size 1.7 μm); Column temperature: 40 °C; Injection volume: 5 μL; Mobile phase: 0.1% formic acid aqueous solution and acetonitrile under gradient elution conditions (Table 1).

Mass spectrometer condition: electrospray ion source; Positive ion scanning; Multiple reaction monitoring; Capillary voltage: 3.5 kV; Ion source temperature: 120 °C; Desolvent gas temperature: 380 °C; Cone gas and desolvent gas: nitrogen (purity 99.9%); Collision gas: argon (99.999% purity); Cone gas flow: 50 L/h; Desolvent gas flow: 600 L/h.

#### 2.3.4. Evaluation of Matrix Effect

In the process of mass spectrometry, components such as protein, polysaccharide, lipid, and other components in the matrix co-extracts of biological samples may interfere with the ionization process of the target, leading to the inhibition or enhancement of analytical signals, resulting in ME. In this study, the slope ratio between the linear equations of the matrix matching standard curve of each test substance and the acetonitrile—0.1% formic acid aqueous solution (*v*/*v*, 5:95) standard curve was adopted for the calculation and evaluation of ME: ME = (the slope of the blank matrix standard curve/the slope of the solvent standard curve − 1) × 100%. Positive ME values suggested a matrix enhancement effect, while negative ME values indicated a matrix inhibition effect. ∣ME∣ ≤ 20% signified a weak ME, 20% < ∣ME∣ ≤ 50% signified a medium ME, and ∣ME∣ > 50% signified a strong ME.

#### 2.3.5. Statistical Analysis

All results were presented as the mean ± standard deviation of three or six independent experiments. SPSS 26.0 software was employed to perform variance analysis and minimum significant difference test; *p*-values < 0.05 were considered to be statistically significant. Excel 2010 and Origin 8.0 were used for chart drawing. The error bars of the figures were generated by the values of the standard deviation.

## 3. Results and Analysis

### 3.1. Optimization of Chromatographic Conditions

C_18_ reversed-phase chromatographic column is commonly used for chromatographic separation of MALs. The basic tertiary amine groups present in the MAL molecules can produce a nonspecific interaction with the residual silyl-hydroxyl groups in the stationary phase of the chromatographic column, resulting in a peak tailing on the ordinary C_18_ column [43]. Since the BEH C_18_ column has no residual silanol groups in its stationary phase, the peak shape can be improved with wider pH tolerance when it is used as the chromatographic column with capped ends. An effective separation of 10 MALs was achieved by using the BEH C_18_ column (length of 2.1 mm × 100 mm and particle size of 1.7 μm), with a good peak shape and high signal-to-noise ratio (S/N). Therefore, the Acquity UPLC C_18_ column (2.1 mm × 100 mm, 1.7 μm) was selected as the separation column.

**Figure 2:**
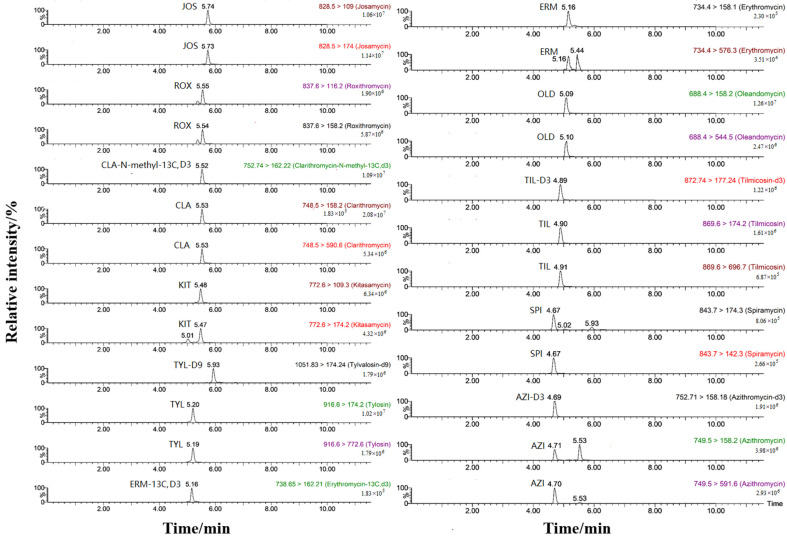
MRM chromatograms of 20 ng/L mixed standard solutions of 10 MALs (the five internal standards were added at a concentration of 5 ng/mL).

The efficiency of methanol and acetonitrile in separating the organic phase was compared. It was found that acetonitrile, a strong polar elution solvent, could shorten the retention time of each analyte by 1–2 min, with a narrower chromatographic peak and higher mass spectrum response signal [44]. To further improve the ionization efficiency of mass spectrometry and the chromatographic peak shape, the effects of adding formic acid or ammonium acetate in the aqueous phase were compared [45,46]. The results demonstrated that when 0.1% formic acid was added to the aqueous phase, the peak retention time of each analyte was shortened, and the peak shape became narrower, suggesting that the addition of formic acid improved the ionization efficiency of MALs as alkaline targets and improved the signal response. Therefore, the response signal value of azithromycin, spiramycin, and tilmicosin nearly doubled. As a result, acetonitrile and 0.1% formic acid aqueous solution were finally selected as mobile phases, and 10 MALs and five internal standards could achieve good chromatographic separation. MRM chromatograms of mixed standard solutions of 10 MALs with a mass concentration of 20 ng/L under the optimized conditions are shown in Figure 2.

### 3.2. Selection of Mass Spectrometry Conditions

Since MALs are weak basic compounds, the positive ion scanning mode (ESI^+^) of electrospray is suitable. The standard solution of a single macrolide (mass concentration was 1.0 μg/mL) was directly injected at a flow rate of 10 μL/min to optimize the acquisition parameters of the mass spectrometer. First, the primary mass spectrometry scanning (precursor ion scanning) was carried out after adjusting the ion source temperature, desolvent gas temperature, capillary voltage, and cone voltage to obtain the [M + H]^+^ molecular ion peak of each analyte. The product ions (product ion scanning) were obtained by adjusting the collision energy. The two ions with high abundance and minimal interference were selected as quantitative and qualitative, respectively. The optimized ion mass spectrometry response was achieved through parameter optimization. The precursor ions of the 10 MALs, five internal standards, two product ions, and the optimal mass spectrum parameters after optimization are shown in Table 2.

### 3.3. Optimization of Pre-Treatment Conditions

#### 3.3.1. Optimization of Extraction Conditions

**Figure 3:**
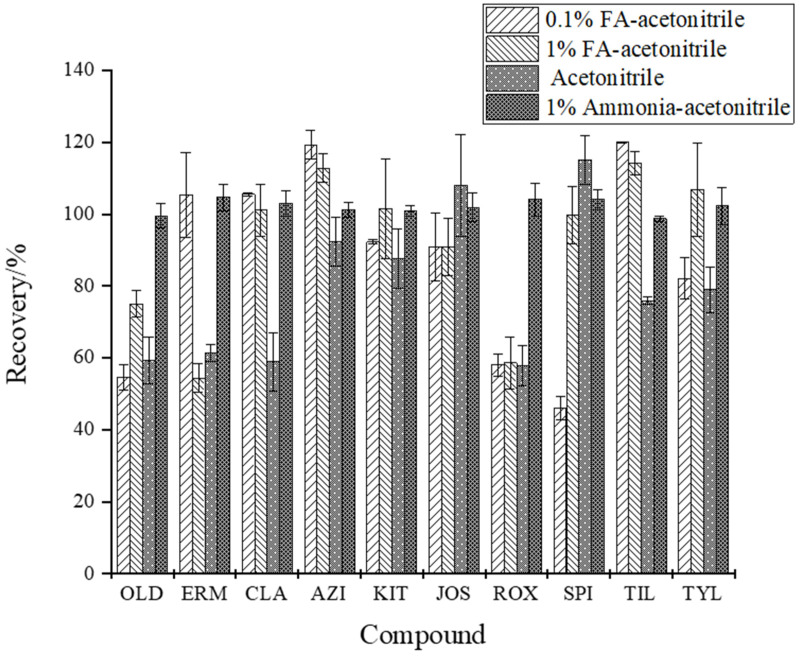
Effects of extraction solvent on the recovery of the 10 MALs (*n* = 3).

Because the MALs detected in this study have weak alkalinity and are soluble in fats, methanol and acetonitrile extraction efficiency on the blank samples of *Carassius auratus* were compared by adding 20 μg/kg of 10 mixed MALs standard solutions. The results showed that the extraction rate of 10 MALs by acetonitrile was higher than by methanol, which may be attributed to the low solubility of acetonitrile to sugars, fats, and other impurities in the samples and the denaturation and precipitation effects of acetonitrile on proteins. Using acetonitrile for extraction reduces the interference of other impurities [47]. We compared the efficiency of four extraction solvents, including pure acetonitrile, 1% acidified acetonitrile, 0.1% acidified acetonitrile, and 1% ammoniated acetonitrile in extracting the 10 MALs in aquatic products. As shown in Figure 3, the results indicated that the recoveries of ERM, CLA, AZI, and TIL were significantly higher with 0.1% formic acid (*p* < 0.05), while those of OLD, ERM, CLA, AZI, KIT, TIL, and TYL were significantly higher with 1% formic acid (*p* < 0.05). Meanwhile, 1% ammonia slightly reduced the recoveries of JOS and SPI (*p* > 0.05) but significantly increased those of OLD, ERM, CLA, AZI, KIT, TIL, and TYL (*p* < 0.05). The recoveries of OLD, ERM, CLA, ROX, and SPI when acetonitrile, 0.1% acidified acetonitrile, and 1% acidified acetonitrile were used for extraction were less than 60%, but those of all the 10 MALs ranged from 98.7% to 104.6% when 1% ammonia acetonitrile was used. According to the p*K*_a_ value of MALs, a weak alkaline environment is more favorable for drug extraction, and this may be because a weak alkaline environment inhibits the molecular ionization of MALs, and molecular compounds have better solubility in organic solvents [48]. The extraction solution with a higher pH can block the hydrogen bonding between MALs and the matrix surface, facilitating the separation of the target compound from the matrix. In addition, since the glycoside bond in the molecule is easy to hydrolyze under acidic conditions [49], MALs are more stable in neutral or weakly alkaline environments. For example, erythromycin is easily converted into dehydrated erythromycin in acidic environments [42]. The present study also revealed that after the formic acid concentration increased from 0.1% to 1%, the CLA, AZI, and TIL recoveries decreased (*p* > 0.05). In particular, ERM decreased significantly from 105.4% to 54.4%. In conclusion, 1% ammonia–acetonitrile was the best extraction solvent for the 10 MALs in aquatic products.

#### 3.3.2. Selection of Purifying Agents

**Figure 4:**
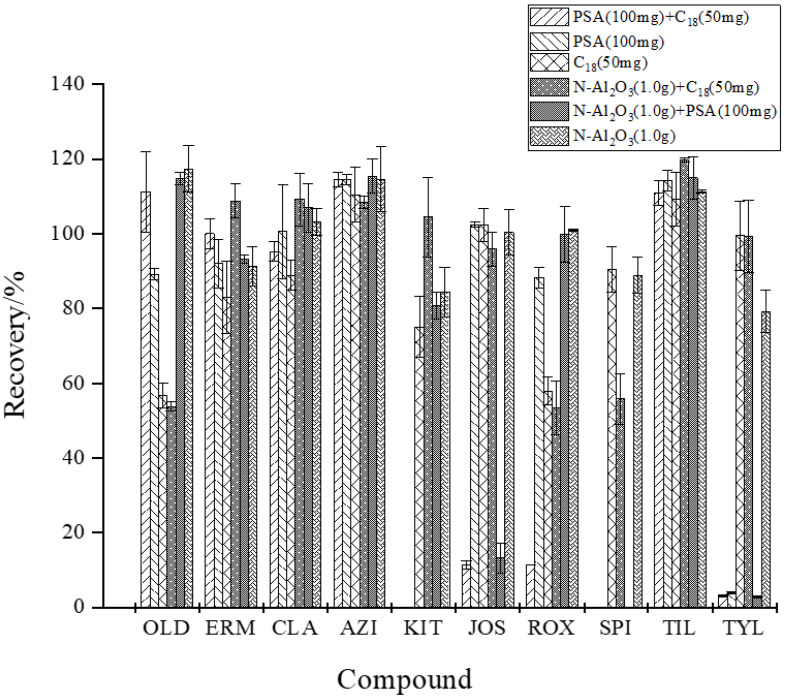
The efficacy of different purifying agents on the recovery of the 10 MALs (*n* = 3).

Considering the complexity of the matrix composition of aquatic products and the molecular structure of MALs, the purification effects of three adsorbents, PSA, C_18,_ and N-Al_2_O_3,_ were compared to the addition of 20 μg/kg in blank crucian carp. Because of the high water content in fish meat, 1.0 g anhydrous MgSO_4_ was also added at the same time as a dispersive solid-phase extractant to reduce the corresponding interferences and facilitate the subsequent concentration operation by nitrogen blowing [50]. As shown in Figure 4, when C_18_ was used as the adsorbent alone, the recoveries of OLD and ROX were only 56.8% and 57.9%, respectively. When PSA was used alone, KIT and SPI were undetectable, and the recovery of TYL was only 3.85%. When PSA+C_18_ was used, the recovery was even worse than those of TYL, ROX, and JOS, only reaching 3.17%, 11.4%, and 11.4%, respectively, while KIT and SPI could not be detected. When N-Al_2_O_3_ was used alone, the recoveries of 10 MALs were 79.3~117.3%, but when N-Al_2_O_3_+C_18_ was added, the recoveries of OLD, ROX, and SPI decreased to 53.8%, 53.4%, and 55.8%, respectively. When N-Al_2_O_3_+PSA was used, SPI could not be detected, and the recoveries of TYL and JOS decreased to 2.83% and 13.3%, respectively. PSA is usually used to remove polar interfering substances such as pigments, carbohydrates, and organic acids, while C_18_ removes non-polar interfering substances such as fats [51]. However, its residual silyl hydroxyl can easily produce a nonspecific effect with polar targets, which leads to a strong adsorption and retention capacity of C_18_ for OLD and ROX and of PSA for KIT, SPI, and TYL, resulting in recovery loss. Thus, using N-Al_2_O_3_ alone can improve the purification of the 10 MALs.

**Figure 5:**
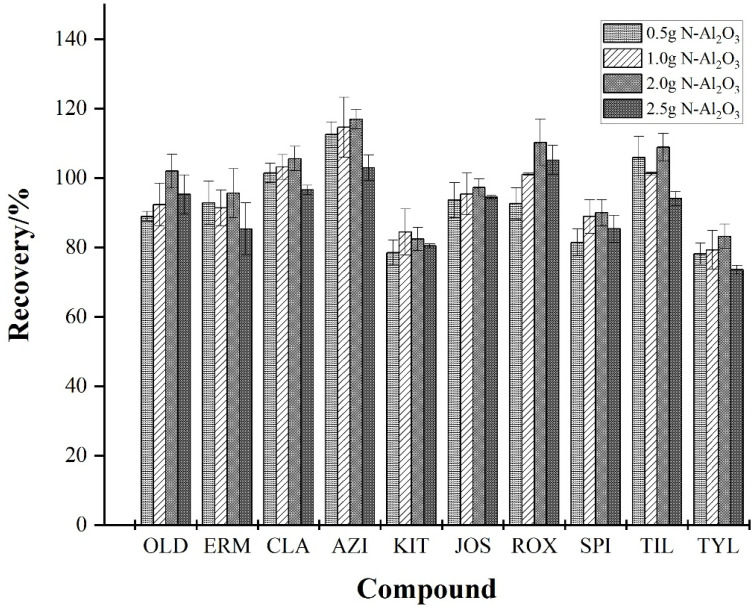
Effect of different N-Al_2_O_3_ contents on the recovery of the 10 MALs (*n* = 3).

The effects of different N-Al_2_O_3_ contents (0.5 g, 1.0 g, 2.0 g, and 2.5 g) on the recovery of MALs were also assessed. As shown in Figure 5, the recovery of most target substances increased with the increase in N-Al_2_O_3_ content until the content reached 2.0 g, beyond which the recovery started decreasing. This could be because the adsorbent not only adsorbs impurities but also targets MALs, and excessive use of adsorbent causes the loss of target substances. Therefore, 2.0 g N-Al_2_O_3_ was used in the subsequent experiments.

### 3.4. Evaluation of the Matrix Effect

**Figure 6:**
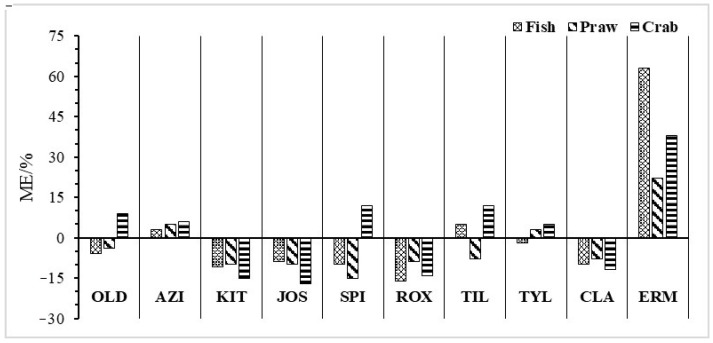
The ME in the 10 MALs in 3 kinds of matrix samples.

Due to the strong binding tendency of the tertiary amine group in MALS molecules with polysaccharides and protein, a strong ME appears on the mass spectrometer. Sample dilution (10 mL was taken from 50-mL sample extract solution for subsequent purification) in the pre-treatment process reduces some matrix interference. On this basis, the ME of the whole sample pre-treatment process of MAL determination in three different fish, shrimp, and crab samples was further evaluated (as shown in Figure 6). Except for ERY, the MEs of nine MALs in three aquatic products were all less than 20%, indicating a weak ME. ME was very strong for ERY in fish tissues (63%), and a moderate ME was observed for shrimp and crab tissues (22% and 38%, respectively). The above results indicated that the pre-treatment method in this study could effectively reduce the interferences caused by polysaccharides, proteins, organic acids, and pigments in the samples. In order to further reduce the effect of ME on the results, isotopically labeled compounds of MALs were selected as internal standards, which were for quantification analysis to eliminate ME and make the quantification more accurate. Because isotope markers are generally expensive and some isotopically labeled compounds of MALs are not currently sold on the market, one isotope marker can be used as a common internal standard for multiple MALs compared to similar molecular structures and molecular weights. According to the comparison of the recovery rate after internal standard correction, the internal standards for the quantification of 10 MALs were listed in Table 2. After quantification with the selected internal standards, the recoveries of 10 MALs could meet the requirements of chemical analysis.

### 3.5. Linearity Range, Limit of Detection, and Limit of Quantification

**Figure 7:**
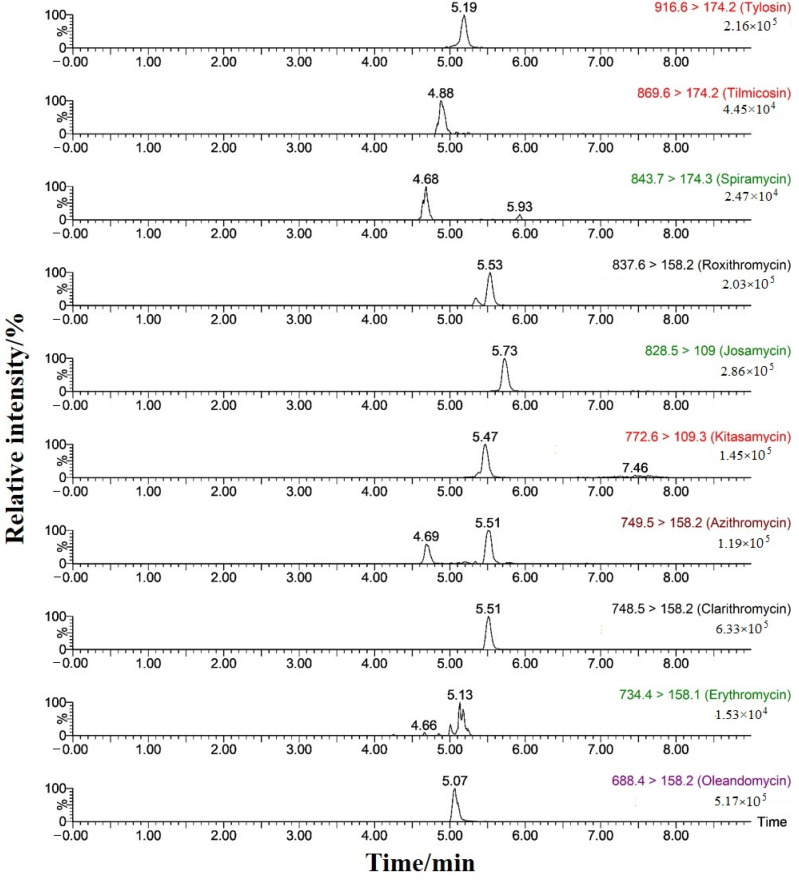
Extracted chromatograms of quantitative ions of 10 MALs at the LOD spiked concentration.

Appropriate amounts of mixtures comprising an external standard solution of 10 MALs and five mixed internal standard solutions were diluted with acetonitrile–0.1% formic acid aqueous solution (*v*/*v*, 5:95) to form a series of solutions (internal standard concentration of 5.0 μg/L) with the mass concentrations of 1.0, 2.0, 5.0, 10.0, 20.0, 50.0, and 100 μg/L. UPLC-MS/MS determination was performed according to the conditions in Section 2.3.3. The ratio Y of each analyte peak area to the internal standard peak area was taken as the ordinate, and the mass concentration X (μg/L) of each analyte was used as the abscissa for linear regression of the calibration curve (1/X as the applied weighting factor). The results showed that within the 1–100 μg/L range, 10 MALs displayed a good linear relationship, with a linear determination coefficient *R*^2^ ≥ 0.99. By measuring the ratio of S/N at the retention time of each MAL, the limit of detection (LOD) and the limit of quantification (LOQ) of the proposed method were calculated using 3 times and 10 times the S/N. The LOD of 10 MALs was 0.25–0.50 μg/kg, and the LOQ was 0.50–1.00 μg/kg (as shown in Table 3). The interference of matrix impurities near the retention time of each target was small, indicating that this method has strong selectivity for target MALs. MRM chromatograms of 10 MALs at the LOD addition concentrations are shown in Figure 7.

### 3.6. Recovery and Precision

Mixed standard solutions of 10 MALs were added into the three blank aquatic samples of *Carassius auratus*, *Litopenaeus vannamei*, and *Portunus trituberculatus* at three levels (1.0, 5.0, and 50.0 μg/kg) over three days with six replicates each day, and then the samples were extracted, purified, and determined according to the current method. Accuracy was expressed as an average recovery percentage for each level and calculated as the ratio between the measured value and the corresponding spiked level, while intra-day precision (*n* = 6) and inter-day precision (*n* = 6, 3d) were calculated as the data’s relative standard deviation (RSD). As shown in Table 4, the average recovery rate of the 10 MALs at the concentration of 1.0 to 50.0 μg/kg was 83.1–116.6%, and the intra-day precision and inter-day precision were 3.7–13.8%, indicating that this method is highly accurate and has a good precision, which meets the requirements for the analysis of MAL residues in aquatic products.

The results for the comparison of our method and the HPLC-MS/MS method reported in other studies for the detection of MAL residues in aquatic products are shown in Table 5. Although the determination sensitivity was not as good as that reported in the Refs. [52,53,54], the current method had obvious advantages in detecting broader MAL spectra and applying more matrix types, which met the simultaneous quantitative analysis of 10 MALs; considering the other studies, this method had higher determination accuracy and sensitivity. DSPE was used for sample purification, and this pre-treatment process is simpler, faster, and cheaper. Notably, combining sample dilution and internal standard correction quantification reduced further ME interference and increased the accuracy and reliability of the results.

### 3.7. Actual Sample Determination

**Figure 8:**
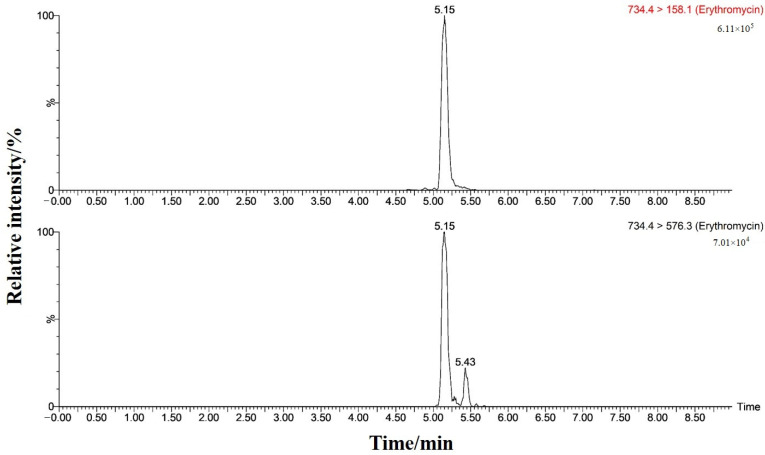
MRM chromatograms of samples in which ERM was detected (20.5 μg/kg).

A total of 60 aquatic samples, including *Ophiocephalus argus*, *Ctenopharyngodon idella*, *Carassius auratus*, *Hypophthalmichthys molitrix*, *Siniperca chuatsi*, *Litopenaeus vannamei*, and *Pseudosciaena crocea*, were collected from the market, pre-treated, and analyzed by UPLC-MS/MS according to the method established. The results showed that the target residue was detected in one *Carassius auratus* sample. The deviation of ERM’s retention time between the positive sample and the standard solution was within 5%, and the abundance ratio of the qualitative ion to the quantitative ion in the test sample was consistent with that of the ERM standard, indicating that the sample was confirmed to be detected by ERM, and the quantitative value was 20.5 μg/kg. The MRM chromatograms of the qualitative and quantitative ions are shown in Figure 8. Overall, the results showed that our method can detect MALs in actual aquatic samples.

## 4. Conclusions

A highly accurate and sensitive UPLC-MS/MS method for determining 10 MAL residues in aquatic products through N-Al_2_O_3_ matrix DSPE purification for sample pre-treatment was developed. Under the optimized conditions of chromatographic separation and mass spectrometry analysis, the analysis of 10 MALs could be completed within 10 min. Compared to the reported analysis methods for MALs in aquatic products, the current method significantly shortened the pre-treatment time, and the use of isotopically labeled compounds as internal standards for quantification could reduce the matrix effect and improve quantitative accuracy. The validation results demonstrated that our method had low LOD and LOQ for 10 MALs, in addition to being accurate and reproducible. Overall, our method is suitable for detecting 10 MALs in aquatic products.

## Figures and Tables

**Table 1:** Mobile phase gradient elution conditions.

Time (min)	Flow (mL/min)	0.1% Formic Acid/%	Acetonitrile/%
0	0.2	95.0	5.0
2	0.2	95.0	5.0
6	0.2	5.0	95.0
8	0.2	95.0	5.0
10	0.2	95.0	5.0

**Table 2:** MRM condition of the 10 MALs and the five internal standards.

Compound	Precursor Ion (m/z)	Product Ion (m/z)	Cone Voltage (V)	Collision Energy (eV)	Internal Standard
Oleandomycin (OLD)	688.4	158.2 *	28	28	ERM-^13^C, D_3_
		544.5		20	
Erythromycin (ERM)	734.0	158.1 *	25	32	ERM-^13^C, D_3_
		576.3		15	
Clarithromycin (CLA)	748.5	158.2 *	12	24	CLA-N-methyl-^13^C, D_3_
		590.6		16	
Azithromycin (AZI)	749.5	158.2 *	26	38	AZI-D_3_
		591.6		28	
Kitasamycin (KIT)	772.6	109.3 *	28	40	AZI-D_3_
		174.2		35	
Josamycin (JOS)	828.5	109.0 *	29	40	CLA-N-methyl-^13^C, D_3_
		174.0		32	
Roxithromycin (ROX)	837.6	116.2	14	44	CLA-N-methyl-^13^C, D_3_
		158.2 *		30	
Spiramycin (SPI)	843.7	142.2	20	35	TIL-D_3_
		174.3 *		37	
Tilmicosin (TIL)	869.6	174.2 *	22	42	TIL-D_3_
		696.7		42	
Tylosin (TYL)	916.6	174.2 *	70	44	TYL-D_9_
		772.6		40	
Erythromycin-13C, D3 (ERM-^13^C, D_3)_	738.65	162.2	48	28	/
Azithromycin-D_3 (_AZI-D_3)_	752.71	158.18	68	38	/
Clarithromycin-N-methyl-13C, D3 (CLA-N-methyl-^13^C, D_3)_	752.74	162.22	52	22	/
Timicosin-D_3 (_TIL-D_3)_	872.74	177.24	70	44	/
Tylosin-D_9 (_TYL-D_9)_	1051.83	174.24	36	40	/

Note: * is a quantitative ion pair.

**Table 3:** Linear equation, determination coefficient, LOD, and LOQ of the 10 MALs.

Compound	Linear Equation	Determination Coefficient	LOD (μg/kg)	LOQ (μg/kg)
OLD	Y = 6.3599X + 13.1310	*R*^2^ = 0.9939	0.25	0.50
ERM	Y = 0.6857X + 0.0630	*R*^2^ = 0.9964	0.50	1.00
CLA	Y = 0.8238X + 0.7683	*R*^2^ = 0.9951	0.50	1.00
AZI	Y = 0.6307X + 1.3856	*R*^2^ = 0.9992	0.50	1.00
KIT	Y = 0.2365X + 0.2288	*R*^2^ = 0.9979	0.50	1.00
JOS	Y = 0.4926X + 0.2954	*R*^2^ = 0.9983	0.50	1.00
ROX	Y = 2.5191X + 5.8606	*R*^2^ = 0.9957	0.25	0.50
SPI	Y = 0.3657X + 0.9069	*R*^2^ = 0.9931	0.50	1.00
TIL	Y = 0.6663X + 1.3854	*R*^2^ = 0.9978	0.50	1.00
TYL	Y = 0.5362X + 0.3864	*R*^2^ = 0.9981	0.50	1.00

**Table 4:** The recovery and precision of UPLC-MS/MS in detecting MALs added in samples.

Analyte	Spiked Levels (μg/kg)	*Carassius auratus*			*Litopenaeus vannamei*			*Portunus trituberculatus*		
		Recovery (%)	Intra-Day RSD (%)	Inter-Day RSD (%)	Recovery (%)	Intra-Day RSD (%)	Inter-Day RSD (%)	Recovery (%)	Intra-Day RSD (%)	Inter-Day RSD (%)
OLD	1	109.7	7.8	10.2	98.2	4.4	5.5	88.6	9.4	11.6
	5	103.2	8.9	11.3	88.4	3.9	4.2	86.1	8.6	9.1
	50	112.4	9.2	9.7	97.8	4.5	5.1	88.5	7.9	8.7
ERM	1	93.9	6.5	4.3	104.9	5.8	7.6	98.0	10.5	9.8
	5	83.2	7.6	9.2	95.8	8.7	10.6	97.7	4.6	4.9
	50	103.3	8.2	10.3	106.6	10.2	9.4	96.7	9.1	11.4
CLA	1	116.6	7.5	8.3	108.9	3.7	4.4	106.1	7.2	6.1
	5	95.3	9.5	12.1	94.9	8.6	10.2	85.8	8.1	9.4
	50	110.1	9.8	11.4	102.0	5.7	7.2	101.7	5.4	4.8
AZI	1	106.7	8.6	9.3	99.7	4.6	5.7	89.0	5.2	4.7
	5	103.6	10.2	13.8	97.4	5.8	4.4	90.8	8.5	8.0
	50	115.9	7.8	11.2	101.9	6.5	5.3	105.6	7.3	6.7
KIT	1	88.7	7.6	6.5	85.6	8.0	9.5	96.8	6.8	7.6
	5	88.9	5.3	4.2	87.5	8.9	9.3	83.8	4.2	4.6
	50	84.4	7.8	9.0	88.5	5.7	5.4	83.7	5.2	4.8
JOS	1	105.3	4.2	6.0	104.0	8.6	12.6	112.6	4.5	5.3
	5	91.0	5.1	5.9	96.5	5.6	8.6	94.2	5.7	6.9
	50	104.9	7.5	8.4	93.0	6.7	7.3	102.2	7.3	8.9
ROX	1	97.4	4.8	4.2	101.8	9.2	8.5	88.0	6.2	7.1
	5	97.0	8.2	9.9	97.2	4.8	5.4	94.7	5.2	4.9
	50	114.1	7.8	9.2	104.6	5.2	4.7	92.1	7.2	6.7
SPI	1	93.5	8.2	10.0	94.0	4.8	6.1	103.3	5.9	6.7
	5	86.3	5.5	4.2	92.7	7.2	8.3	83.5	5.1	4.6
	50	86.5	9.8	12.6	91.8	6.8	8.0	94.6	7.8	11.7
TIL	1	108.2	6.5	7.3	103.2	7.3	8.9	114.8	5.7	7.1
	5	97.6	5.3	4.7	97.6	5.2	4.0	102.7	4.8	5.1
	50	112.7	9.4	11.5	102.8	5.1	6.7	110.1	5.2	4.7
TYL	1	83.1	6.8	9.1	89.1	9.5	11.4	90.4	5.9	7.6
	5	86.5	4.5	3.9	92.5	7.8	10.5	88.0	6.2	8.5
	50	87.3	6.8	8.6	85.8	6.2	8.3	87.4	6.7	7.3

**Table 5:** Comparison of HPLC-MS/MS methods for determining MAL residues in aquatic products.

Matrix	Extraction	MALs	Purification	Quantification	Recovery (%)	LOD (μg/kg)	LOQ (μg/kg)	Literature
Fish	EDTA + acetonitrile	ERM	Direct dilution of sample extract	Matrix matching external standard curve	89.2~119.5	1.0	3.0	[55]
Fish	Acetonitrile: water	AZI, CLA, ERM, ROX, and TYL	Strata-X SPE	Internal standard curve	31~68	0.004~0.054	0.013~0.18	[52]
Shrimp and fish	EDTA + acetonitrile	AZI, CLA, and ERM	Defatting by n-hexane liquid-liquid extraction	Matrix matching external standard curve	92.0~99.2	0.053–0.417	0.159~1.251	[53]
Fish	EDTA + ethylacetate	CLA and ROX	Oasis HLB SPE	Matrix matching & internal standard curve	80.1~90.5	0.01~0.22	0.05~0.7	[54]
Shrimp	EDTA + acetonitrile	ERM, TIL, JOS, TYL, and SPI	Defatting by n-hexane liquid-liquid extraction	Matrix matching external standard curve	74.3~111.1	2.0	5.0	[56]
Fish, shrimp, crab, and shellfish	Acetonitrile	OLD, ERM, CLA, AZI, JOS, KIT, SPI, TIL, and TYL	Defatting by n-hexane liquid-liquid extraction +N-Al_2_O_3_ SPE	Matrix matching external standard curve	70~120	1.0	2.0–4.0	[57]
Fish	Methanol (pressurized liquid extraction)	ERM, JOS, ROX, SPI, TIL, OLD, TYL	Al_2_O_3_ DSPE	Matrix-matching external standard curve	66~91	15	25~50	[58]
Fish, shrimp, crab, and shellfish	Acetonitrile-ammonia	OLD, ERM, CLA, AZI, JOS, KIT, SPI, TIL, TYL, and ROX	N-Al_2_O_3_ DSPE	Internal standard curve	83.1~116.6%	0.25–0.50	0.50–1.00	Present method

## Data Availability

The original contributions presented in the study are included in the article, further inquiries can be directed to the corresponding author.
